# Ultrafast Coherent Hole Injection at the Interface between CuSCN and Polymer PM6 Using Femtosecond Mid-Infrared Spectroscopy

**DOI:** 10.1021/acsami.4c01156

**Published:** 2024-04-04

**Authors:** George Healing, Issatay Nadinov, Wisnu Tantyo Hadmojo, Jun Yin, Simil Thomas, Osman M. Bakr, Husam N. Alshareef, Thomas D. Anthopoulos, Omar F. Mohammed

**Affiliations:** † Advanced Membranes and Porous Materials Center, Division of Physical Science and Engineering, King Abdullah University of Science and Technology, Thuwal 23955-6900, Kingdom of Saudi Arabia; ‡ KAUST Catalysis Center, Division of Physical Sciences and Engineering, King Abdullah University of Science and Technology, Thuwal 23955-6900, Kingdom of Saudi Arabia; § KAUST Solar Center, Physical Science and Engineering Division, King Abdullah University of Science and Technology (KAUST), Thuwal 23955-6900, Kingdom of Saudi Arabia; ∥ Department of Applied Physics, The Hong Kong Polytechnic University, Hung Hom, Kowloon, Hong Kong; ⊥ Materials Science and Engineering, Physical Science and Engineering Division, King Abdullah University of Science and Technology (KAUST), Thuwal 23955-6900, Kingdom of Saudi Arabia

**Keywords:** CuSCN, PM6, ultrafast hole injection, hole transport layer, photovoltaics, fs mid-IR

## Abstract

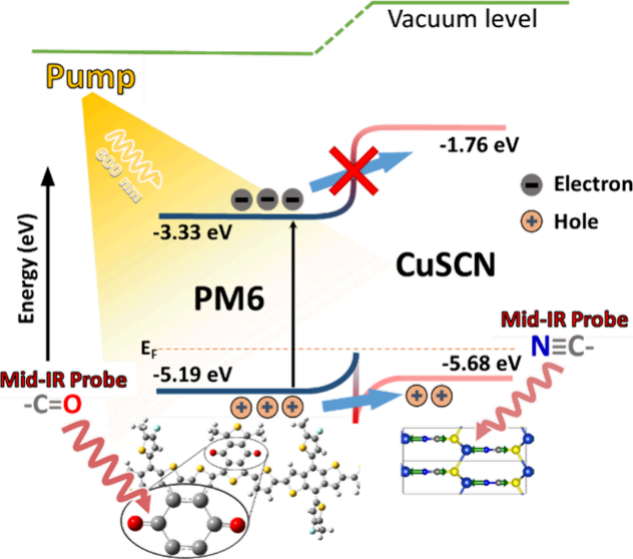

Tracking the dynamics of ultrafast hole injection into copper thiocyanate (CuSCN) at the interface can be experimentally challenging. These challenges include restrictions in accessing the ultraviolet spectral range through transient electronic spectroscopy, where the absorption spectrum of CuSCN is located. Time-resolved vibrational spectroscopy solves this problem by tracking marker modes at specific frequencies and allowing direct access to dynamical information at the molecular level at donor–acceptor interfaces in real time. This study uses photoabsorber PM6 (poly[(2,6-(4,8-bis(5-(2-ethylhexyl-3-fluoro)thiophen-2-yl)-benzo[1,2-*b*:4,5-*b*′]dithiophene))-*alt*-(5,5-(1′,3′-di-2-thienyl-5′,7′-bis(2-ethylhexyl)-benzo[1′,2′-*c*:4′,5′-*c*′]dithiophene-4,8-dione))]) as a model system to explore and decipher the hole transfer dynamics of CuSCN using femtosecond (fs) mid-infrared (IR) spectroscopy. The time-resolved results indicate that excited PM6 exhibits a sharp vibrational mode at 1599 cm^–1^ attributed to the carbonyl group, matching the predicted frequency position obtained from time-dependent density functional theory (DFT) calculations. The fs mid-IR spectroscopy demonstrates a fast formation (<168 fs) and blue spectral shift of the CN stretching vibration from 2118 cm^–1^ for CuSCN alone to 2180 cm^–1^ for PM6/CuSCN, confirming the hole transfer from PM6 to CuSCN. The short interfacial distance and high frontier orbital delocalization obtained from the interfacial DFT models support a coherent and ultrafast regime for hole transfer. These results provide direct evidence for hole injection at the interface of CuSCN for the first time using femtosecond mid-IR spectroscopy and serve as a new investigative approach for interfacial chemistry and solar cell communities.

## 1 Introduction

Recently, research into copper thiocyanate (CuSCN) as an efficient hole-transporting layer (HTL) has experienced immense growth because it is an effective transporting layer with numerous advantageous properties in optoelectronic and solar-cell devices.^1−4^ Such properties include thermal stability, transparency throughout the visible spectral range, potential scalability due to the availability of relatively inexpensive reagents, and low-temperature solution-based processing.^5−9^ As such, this makes it an excellent candidate as an HTL, favored by the optoelectronic and photovoltaic industries.^10^ Recent studies have attempted to ascertain the dynamics governing its fast and efficient hole-acceptor capability. For instance, Li et al. revealed that carrier relaxation and transfer strongly correlate with the vibrational excitation and relaxation of the CN stretching mode of CuSCN through time-resolved infrared (IR) measurements.^11^ Prior research has also uncovered a temperature dependence in the hole mobility, predominantly triggered by a thermally activated transport mechanism that controls charge carriers at room temperature.^12^

Studying CuSCN and other HTLs in an interfacial arrangement with a corresponding hole donor is necessary to fully understand their role in efficiently extracting and transporting holes. Hole transport dynamics at the interface between CuSCN and an absorber layer have yet to be investigated, partly due to the difficulty of probing the electronic transitions in the hole donor and acceptor layers in the junction. The charge transfer rate at heterojunctions spans a wide range, but the most efficient processes occur on the femtosecond (fs) to picosecond (ps) time scale, necessitating ultrafast pump–probe spectroscopy to investigate their origins. Inevitably, to initiate the hole transfer process, one must selectively excite a photoabsorber and subsequently probe a feature of the transient system to track the spectral dynamics in real time. Run-of-the-mill transient absorption measurements probing in the ultraviolet (UV)-to-visible range might be the first option. However, the transient spectra of many organic and inorganic photovoltaic materials often come with high degrees of complexity associated with the convolution of broad and overlapping electronic states.

Consequently, such complexity detracts from the structural resolution of the process, which can be detrimental to the overall analysis. Furthermore, electron spin-resonance techniques also fall short because they do not have the time resolution to track rapid charge transfer events. The challenge of measurement using femtosecond transient electronic spectroscopies to investigate hole injection in large bandgap semiconductors can be further elaborated as follows. For CuSCN, probing across its large bandgap requires a high-energy probe concomitantly probing an organic donor, which is polymer PM6 in this case. Therefore, the difference spectrum would be a convolution of the spectra from both materials. A probe pulse of mid-IR energy is ideal for selectively measuring vibrational transitions in only CuSCN to accurately indicate hole injection dynamics. By implementing time-resolved UV–vis pump–IR probe spectroscopic measurements, we can examine the dynamics of interfacial hole injection originating from the photoabsorber or the HTL. This approach allows for selectively tracking unique vibrational modes in each material.

The main advantage of the femtosecond mid-IR technique is that it can selectively probe specific frequencies without overlap from electronic transitions. This feature of the technique makes it uniquely suited for independently investigating species on either side of the interface, even at the molecular level. Thus, a frequency was selected for these purposes, corresponding to the CN asymmetric stretch in CuSCN, sufficiently separated from other vibrational modes. Based on the literature review, there are currently no recorded instances of using femtosecond mid-IR spectroscopy to monitor hole injections precisely at organic–inorganic interfaces, to the best of our knowledge. However, numerous examples of ultrafast (i.e., fs time scale) interfacial electron injection studies at the junction between many systems have been measured using the same technique. The case of electron transfer at the interface between a ruthenium sensitizer and nanocrystalline semiconductor in thin films/solutions was reported for the first time by the J. Asbury group using fs mid-IR spectroscopy to simultaneously monitor vibrational marker modes in both materials.^13,14^ Subsequent studies have included organic dyes, such as Coumarin 343.^15^ Monitoring the rise of CN stretching bands at 2040 cm^–1^ for Ru–N3 while adhered to Al_2_O_3_ thin films revealed an electron injection process of <100 fs^13^ and around 50 fs for Ru N3-TiO_2_.^16^ The reporting of such ultrafast time components must remain qualitative, so long as the signal of the marker mode first occurs within the time resolution of the specific measurement.

**Figure 1:**
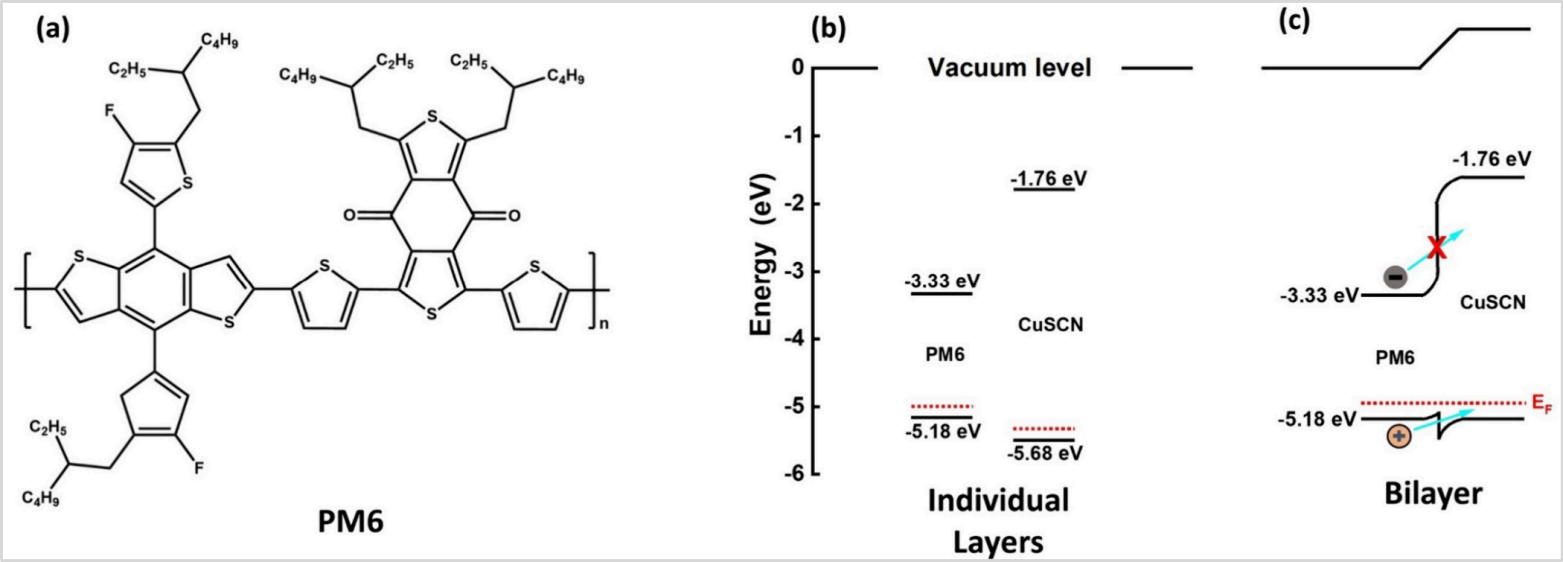
(a) Full chemical structure of a PM6 monomer unit. (b) Energy levels of PM6 and CuSCN before contact and (c) energy levels of PM6 and CuSCN in a bilayer arrangement.

This study uses the polymer PM6 (Figure 1a) as the absorber layer to serve as the hole donor (and concurrent electron acceptor), while CuSCN acts as the hole acceptor. In addition, PM6 was chosen for the following reasons:

(a) In an interfacial arrangement, it exhibits a suitable bandgap alignment with CuSCN as the hole acceptor. (b) It contains the carbonyl group, which manifests a strong vibrational peak in a well-separated spectral range.^17^ (c) Finally, PM6 has typically been used as a donor due to its deep-lying highest occupied molecular orbitals (HOMOs) and relatively large energy gap, resulting in high open-circuit voltage (*V*_OC_) when paired with low-bandgap non-fullerene acceptors in organic solar cells.^18−21^

In contrast, CuSCN is an appropriate choice of hole acceptor in this study because it has a strong CN stretching vibration marker mode in a well-separated spectral range of approximately 2100–2200 cm^–1^.^22,23^ Individually, the energy levels of the HOMO of PM6 and the VBM of CuSCN do not align in a conducive manner to facilitate hole transfer (Figure 1b). However, when brought together in an interfacial arrangement, the generation of an ohmic contact shifts the energy levels (Figure 1c), enabling the desired alignment necessary for hole transfer upon photoexcitation.^24,25^ Forming ohmic contact between the polymer and metal is essential to reduce the energy barrier and minimize the energy loss of organic electronics.^26^ The blue spectral shift of the CN stretching vibration from 2118 cm^–1^ for CuSCN to 2180 cm^–1^ for PM6/CuSCN corroborates this computational study in analyzing the corresponding shift positions and band identification. The kinetic results revealed an ultrafast hole injection from PM6 to CuSCN with a time constant of 168 fs. The ultrafast nature of this transfer implied a coherent charge transfer process between the two materials and necessitated further theoretical support from the density functional theory (DFT) and time-dependent (TD) DFT calculations. Although some studies suggest that separation of charges following coherent transfer may occur too fast to contribute significantly to device performance,^27^ other investigations have demonstrated that the importance of coherent wave functions in influencing the ultrafast separation of charge carriers at the interfaces.^28,29^

## 2 Results

### 2.1 Steady-State and Optical Properties

**Figure 2:**
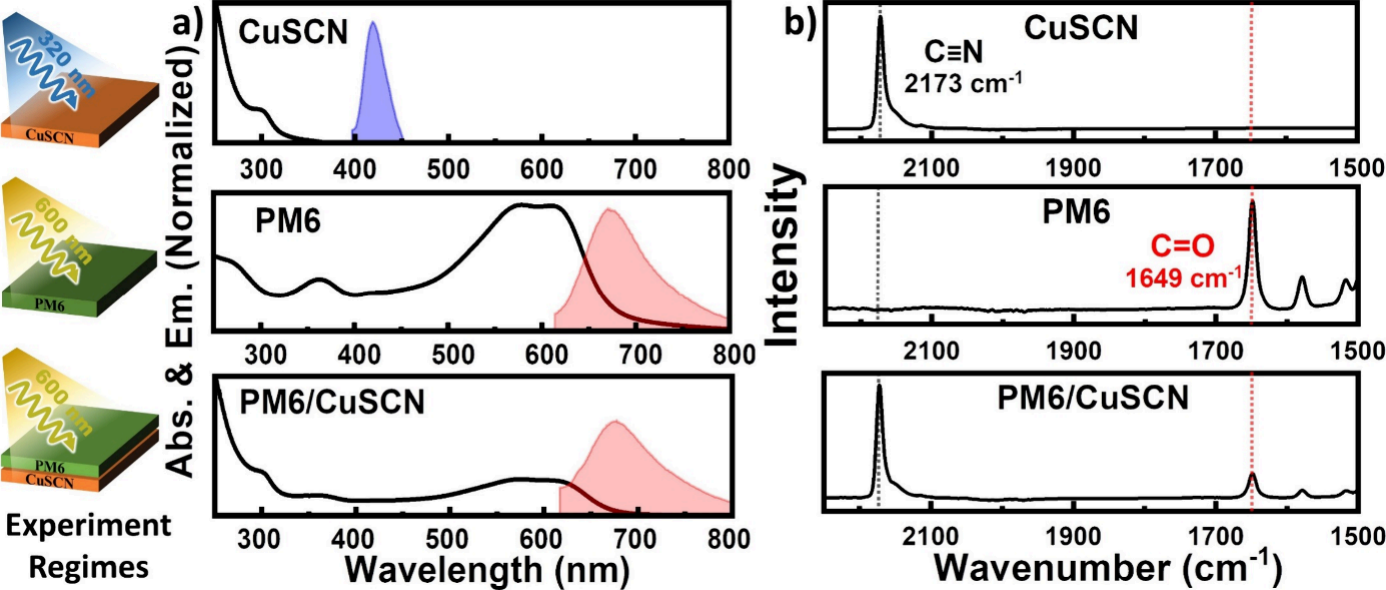
(a) Normalized absorption and emission spectra for the three experimental modes: pure CuSCN excited at 320 nm, pure PM6 excited at 600 nm, and heterojunction PM6/CuSCN excited at 600 nm. (b) Steady-state Fourier transform infrared (FTIR) spectra for each experiment.

Three experimental regimes were established to evaluate the hole injection from the light absorber, PM6, to the CuSCN. Thin films of CuSCN alone are excited directly at 320 nm, but the thin-film PM6 alone and the PM6/CuSCN heterojunction are excited at 600 nm to selectively excite the PM6 layer. Normalized absorption and emission spectra are depicted in Figure 2a. The absorption spectra for CuSCN reveal a characteristic double peak feature around 320 and 260 nm with a tail associated with transitions from band-tail states, frequently observed in CuSCN studies.^25^ These band-tail states consist mainly of native Cu vacancies and morphological imperfections such as grain boundaries that are reported to be approximately 0.2 eV above the valence band maximum (VBM).^3^ The absorption spectrum for PM6 is characterized by two distinct bands: one at 360 nm and another centered at 570 nm. The strong shoulder at 620 nm is attributed to polymer chain aggregates when PM6 is solid.^17^ Following excitation at 600 nm, PM6 exhibits a broad featureless emission centered at 670 nm, often observed in D–A conjugated polymers.^30^ The PM6/CuSCN film produced a superposition of the individual absorption spectra with all previously ascribed absorption peaks in the measurement. Note that no new absorption band was observed in the bilayer. However, normalizing the absorption spectra of both PM6 and PM6/CuSCN reveals slight broadening of the main spectral peak between 450 and 600 nm, which may be attributed to a small distortion of the ground-state geometry of PM6 at the interface with CuSCN (Figure S1). The FTIR spectrum (Figure 2b) for CuSCN reveals an intense peak at 2173 cm^–1^ for the characteristic asymmetric CN stretch. The intense carbonyl peak of PM6 is also observable at 1649 cm^–1^, adjacent to the fingerprint region. The combined experiment reveals the persistence of CN and C=O peaks, as indicated via the dashed black and red lines, respectively. More importantly, the peaks are separated well at over 600 cm^–1^ apart, indicating that both can be followed in the time-resolved measurement without the risk of spectral overlap.

From the Tauc plot analysis (Figure S2b), the direct bandgap energy for CuSCN was 3.92 eV, aligning with the previous electronic bandgap measurements in the literature.^1^ The HOMO levels of PM6 and CuSCN are measured by photoelectron spectroscopy in the air (PESA) and are 5.18 and 5.68 eV, respectively, as demonstrated in Figure S2c,d. The lowest occupied molecular orbitals (LUMO) of PM6 and CuSCN were determined by their relative bandgaps. The energy levels of pristine PM6 and CuSCN are illustrated in Figure 1b. X-ray diffraction characterization studies (Figure S3) identified a crystalline structure for CuSCN and confirmed a β-CuSCN arrangement.^31^

### 2.2 Femtosecond Mid-Infrared Spectroscopy of PM6 and CuSCN

**Figure 3:**
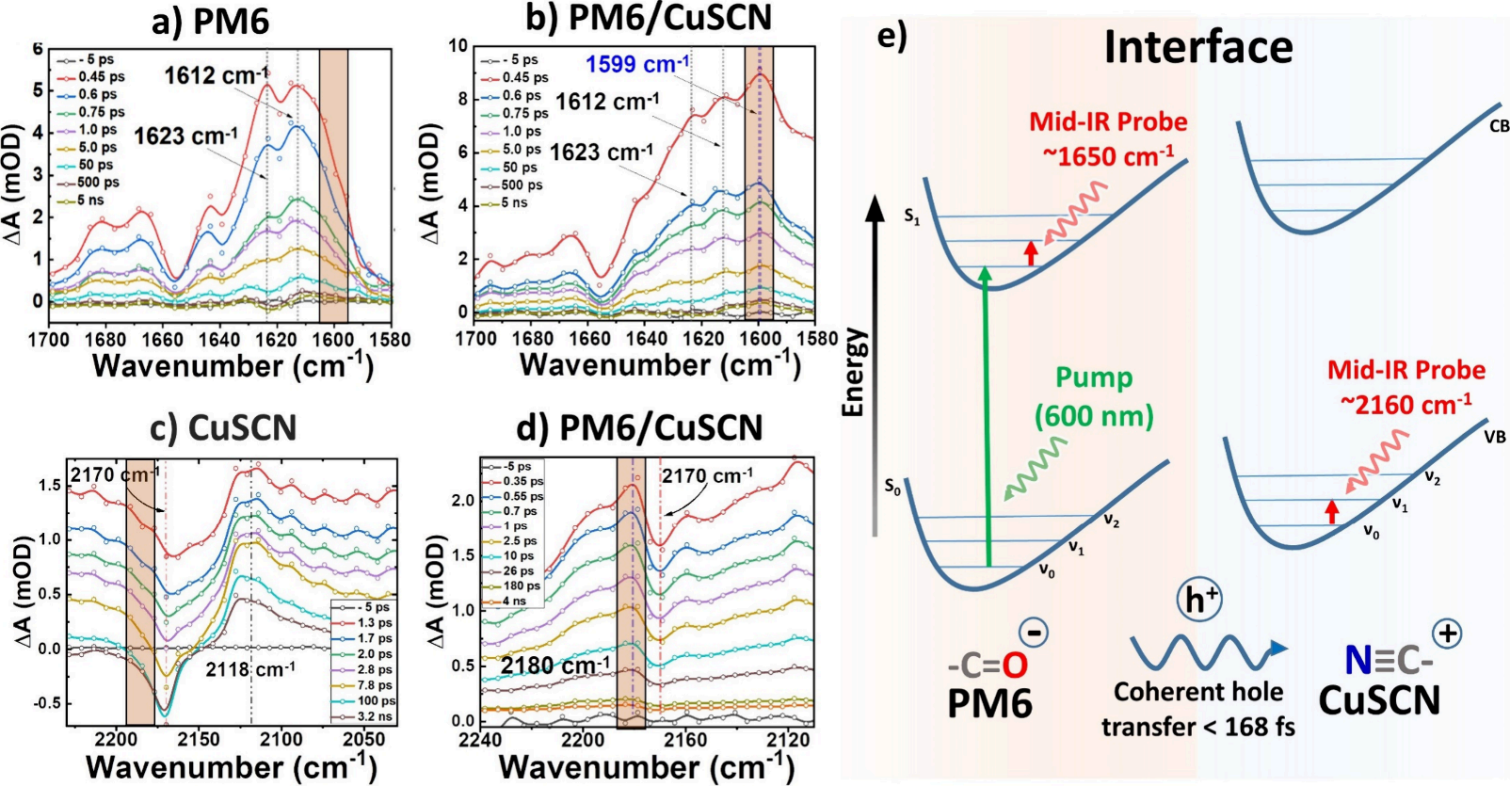
(a, b) Femtosecond mid-infrared (IR) spectroscopy measurements of PM6 and PM6/CuSCN respectively, probing C=O (λ_exc_ = 600 nm). (c) Femtosecond mid-IR spectra for CuSCN (λ_exc_ = 320 nm) and (d) PM6/CuSCN (λ_exc_ = 600 nm), probing the CN asymmetric stretch region. The highlighted regions in (a–d) show the positions of the expected vibrational bands due to hole transfer. (e) Scheme showing the principal vibrational transitions in the fs mid-IR experiment for PM6/CuSCN.

To provide evidence for the hole transfer from PM6 to CuSCN, a series of femtosecond mid-IR measurements were conducted, focusing on probing two key regions. The first region, centered around 1650 cm^–1^, was chosen to probe the C=O vibrational mode present in PM6, whereas the second region around 2160 cm^–1^ primarily targeted the CN asymmetric stretching mode in CuSCN. Figure 3a–d shows the femtosecond mid-IR spectra at various time delays for PM6, CuSCN, and the PM6/CuSCN bilayer. Figures 3a and 3b clearly illustrate the C=O vibrational marker mode of PM6. In contrast, Figures 3c and 3d showcase the distinctive features of the CN asymmetric stretch in CuSCN. To probe the excited-state vibrational mode of CuSCN by itself, an excitation wavelength of 320 nm was used. In order to selectively excite PM6, all other femtosecond mid-IR measurements utilized a 600 nm pump, including the PM6/CuSCN bilayer measurements. It should be noted that the thickness of the PM6 layer was kept at ∼60 nm to ensure consistent transmission of the IR probe throughout the experiment. A scheme showing the principal vibrational transitions in the fs mid-IR measurements is shown in Figure 3e. All contour plots for the associated measurements can be found in the Supporting Information (Figure S4a–d).

**Figure 4:**
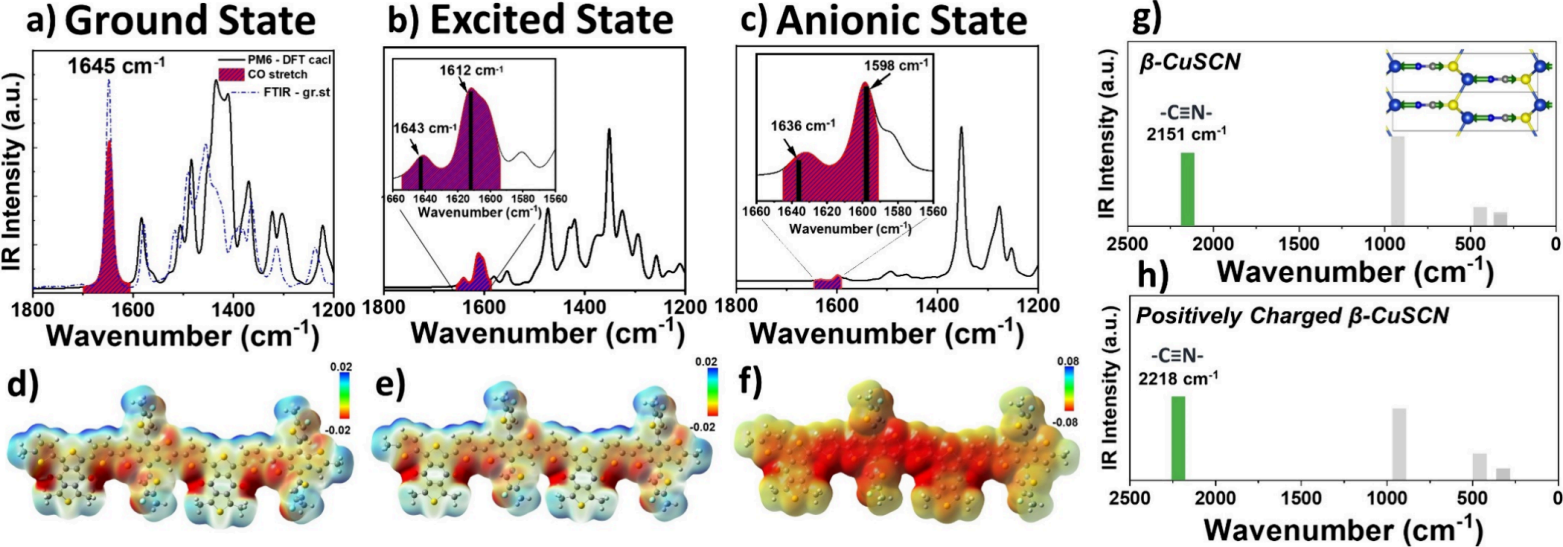
(a–c) Calculated infrared (IR) spectra in the ground, excited, and anionic states, respectively. (d, f) Electrostatic potential maps for PM6 for the respective calculations. (g, h) Calculated IR spectra for CuSCN and positively charged CuSCN.

The femtosecond mid-IR spectra of PM6 (Figure 3a) reveal the ground-state bleach signal at 1654 cm^–1^, closely matching the carbonyl stretch in the FTIR measurement at 1649 cm^–1^ (Figure 2b). The red-shifted excited-state absorption signal of the carbonyl stretch is also evident and is split into two separate bands at 1623 and 1612 cm^–1^. Selectively exciting PM6 in a PM6/CuSCN bilayer produced the femtosecond mid-IR spectra in Figure 3b, which exhibits the same bands as the lone PM6 experiment except for the addition of a new peak at 1599 cm^–1^. The peak position at 1599 cm^–1^ matches well with the DFT-predicted vibration spectrum (Figure 4c) for PM6 when modeled as an anion at 1598 cm^–1^ and represents a new carbonyl stretch band in addition to the split bands at 1623 and 1612 cm^–1^. The presence of a new band when PM6 is layered with CuSCN suggests that interfacial conditions change the charge distribution and, thus, also the frequency of the carbonyl band at the interface.

Carrying out control measurements where CuSCN is excited at 320 nm and probed in the carbonyl region at ∼1640 cm^–1^ shows no discernible vibrational signatures (Figure S5a,b). Therefore, in the bilayer arrangement (PM6/CuSCN), any signal in this carbonyl region must come exclusively from PM6. It was worth noting that even in the absence of CuSCN, the peak at ∼1595 cm^–1^ still remains in PM6 alone as a weak shoulder (Figure 3a) and represents a carbonyl stretch associated with accumulated negative charge. However, exciting PM6 may still result in local trapping of negative charges which can arise due to the naturally disordered microstructure of conjugated organic polymers, such as PM6.^32,33^ Nevertheless, the relative intensity of the 1599 cm^–1^ band relative to others at 1612 and 1623 cm^–1^ points to a large population of these carbonyls in the presence of excess negative charge, which would only be possible following hole injection to CuSCN.

To find the excited-state vibrational signature of the CN asymmetric stretch in CuSCN, a thin film of CuSCN was excited directly at 320 nm and probed in the region of ∼2150 cm^–1^, depicted in Figure 3c. The bleach band at 2170 cm^–1^ represents the 0 → 1 vibrational transition in the ground state, whereas the 2118 cm^–1^ band indicates the 0 → 1 vibrational transition in the excited state. The bleach signal matches the position of the CN asymmetric stretch in the FTIR spectrum (Figure 1b) at 2173 cm^–1^, whereas the excited-state CN band at 2118 cm^–1^ matches previously reported red-shifted excited-state CN asymmetric bands in the literature.^13,22,23^ The large offset in the fs mid-IR spectra for CuSCN in Figure 3c indicates intraband IR absorption by electrons and is a common feature in time-resolved IR measurements for semiconducting materials.^34^ The recovery for these photogenerated carriers is approximately 100 ps as the offset returns to normal, revealing the persistence of the bleach and excited-state absorption signals.

Figure 3d shows the femtosecond mid-IR plot for the PM6/CuSCN bilayer and follows the CN asymmetric stretch of CuSCN following selective excitation of PM6 at 600 nm. For PM6/CuSCN, the bleach signal remained at the same frequency at 2170 cm^–1^. As CuSCN is not directly excited, the bleach signal in Figure 3d necessitates further elaboration. Following hole injection into CuSCN, there is a decrease in the population of neutral CN and a concurrent increase in the population of CN surrounded by positive charges from the injected holes. As a consequence, the neutral CN band absorbs less at 2170 cm^–1^ after pumping, corresponding to a negative signal in the difference spectrum. The positive signal at 2180 cm^–1^ indicates a 0 → 1 vibrational transition of the new CN population surrounded by positive charges. A blue-shift of the CN band in a positively charged CuSCN unit cell was predicted using DFT calculations (see Figure 4g), providing further support for the evaluation of this new vibrational band in the fs mid-IR spectrum and for hole transport in the PM6/CuSCN bilayer. To rule out any contributions of PM6 to the feature at 2180 cm^–1^, control measurements were performed in which PM6 was excited at 600 nm and probed in the CN vibration region at ∼2170 cm^–1^ (Figure S5c,d). Noticeable was the characteristic offset of the vibrational spectrum corresponding to a commonly reported broad polaronic absorption in the mid-IR region for organic semiconductors.^35^ The offset displays no discernible signals, allowing a confident assignment of the 2180 cm^–1^ in Figure 3d for the CN stretching mode of CuSCN due to the hole transfer.

Figure S6 presents the normalized kinetic plots for the carbonyl stretch of PM6 at 1599 cm^–1^ and the CN asymmetric stretch of positively charged CuSCN at 2180 cm^–1^ at early times of up to 1.5 ps. The kinetic fitting of the CN band at 2180 cm^–1^ indicates that the signal maximum was reached after approximately 350 fs and matches the early rise of the carbonyl stretch. The rise of the CN band on this time scale following the selective excitation of PM6 indicates that the hole injection is swift. The kinetics of the CN band can be fit with a triexponential decay consisting of three time constants at 0.3 ns (64%), 16 ns (29%), and ∼4 ns (7%). The fast time component at 0.3 ps is associated with electron–electron scattering processes whereas the 16 ps component is attributed to a possible energy transfer phenomenon between PM6 and the CN stretching mode, both constants found previously by Li et al.^11^ The fast rise of the CN signal associated with the hole injection is obscured within the fitting of the instrument response function (IRF), which is approximately 168 fs for the setup (Figure S7). Therefore, we can only qualitatively ascribe a time constant of <168 fs for the hole transfer event, as it occurred within the temporal profile of the excitation pulse. Worth noting is that the appearance of thermally assisted vibrational transitions via heat accumulation from the pump pulse can introduce potential difficulties in the assignment of specific bands in the fs mid-IR spectrum. However, in this case the possibility of the CN signal arising due to heating by the pump pulse is ruled out due to the sample being placed on a translating stage. This arrangement ensures that each successive pump pulse targets a fresh spot on the sample.

### 2.3 Computational Analysis

The optimization and frequency DFT calculations were conducted to aid in evaluating the excited-state vibrational bands of PM6 and CuSCN. The polymer PM6 was modeled by using two repeating monomer units in the ground, first-excited, and product anionic states. The anionic state simulates the effect of negative charge accumulation on the frequency of the C=O stretch vibrational mode following the transfer of holes to CuSCN. Figure 4 depicts the IR spectra for each calculation and their respective electrostatic potential mapping. In Figure 4a, the calculated ground-state spectrum matches the FTIR spectrum, clearly demonstrating the intense C=O carbonyl stretch at 1645 cm^–1^. The excited-state IR spectrum (Figure 4b) indicates a main red-shifted peak for the C=O stretch at 1612 cm^–1^ with auxiliary peaks at 1643 and 1603 cm^–1^ (highlighted in magenta), associated with local vibrational modes. The main carbonyl stretch peak is red-shifted further to 1598 cm^–1^ (Figure 4c) when PM6 is modeled in the anionic state as the excess negative charge populates the CO π* antibonding molecular orbital, weakening the bond further in the excited state.

Figure 4d–f shows the optimized structures with electrostatic mapping showing the accumulation of the negative charge concentrated primarily along the thiophene backbone. The relative optimized structures without electrostatic mapping are listed in Figure S8. Figure 4g,h shows the β-CuSCN modeled as a neutral and positively charged single unit cell with the latter used to simulate the shift of the asymmetric CN stretch following the hole injection from PM6. The frequency calculations for the β-CuSCN in neutral and positively charged states reveal a 66 cm^–1^ blue-shift of the CN vibrational band, attributed to an increased CN bond order. In the measurements, the femtosecond mid-IR probe of the CN band displayed a similar trend for pure CuSCN and PM6/CuSCN films. The calculations (Figure 4h) predicted a higher frequency for the CN band shift at 2218 cm^–1^ instead of the experimental value of 2180 cm^–1^. This discrepancy of 38 cm^–1^ is most likely due to the pronounced effect of the positive charge/hole on the CN band when modeled as a single-unit cell.^36^ However, from the fs mid-IR spectrum (Figure 3d), we can still successfully attribute the new band at 2180 cm^–1^ to the positively charged interfacial CN vibration, given that it is the primary blue-shifted peak in that spectrum region.

The DFT calculations were for the hole donor and acceptor systems separately; therefore, to obtain further details on the electronic properties at the interface, we optimized PM6 on β-CuSCN using a slab model. To ascertain the correct orientation of PM6 on CuSCN, grazing-incidence wide-angle X-ray scattering (GIWAXS) was performed on CuSCN and PM6/CuSCN samples, shown in Figures S9 and S10. The main difference between CuSCN and CuSCN/PM6 is the appearance of a new peak at 0.2–0.3 A^–1^ in the Qz. This would indicate the presence of PM6 with a face-on orientation on the surface of CuSCN. Furthermore, additional DFT calculations using the same methods reveal a face-on configuration for PM6 is more stable by ∼3.37 eV compared to an edge-on configuration (Figure S11).

**Figure 5:**
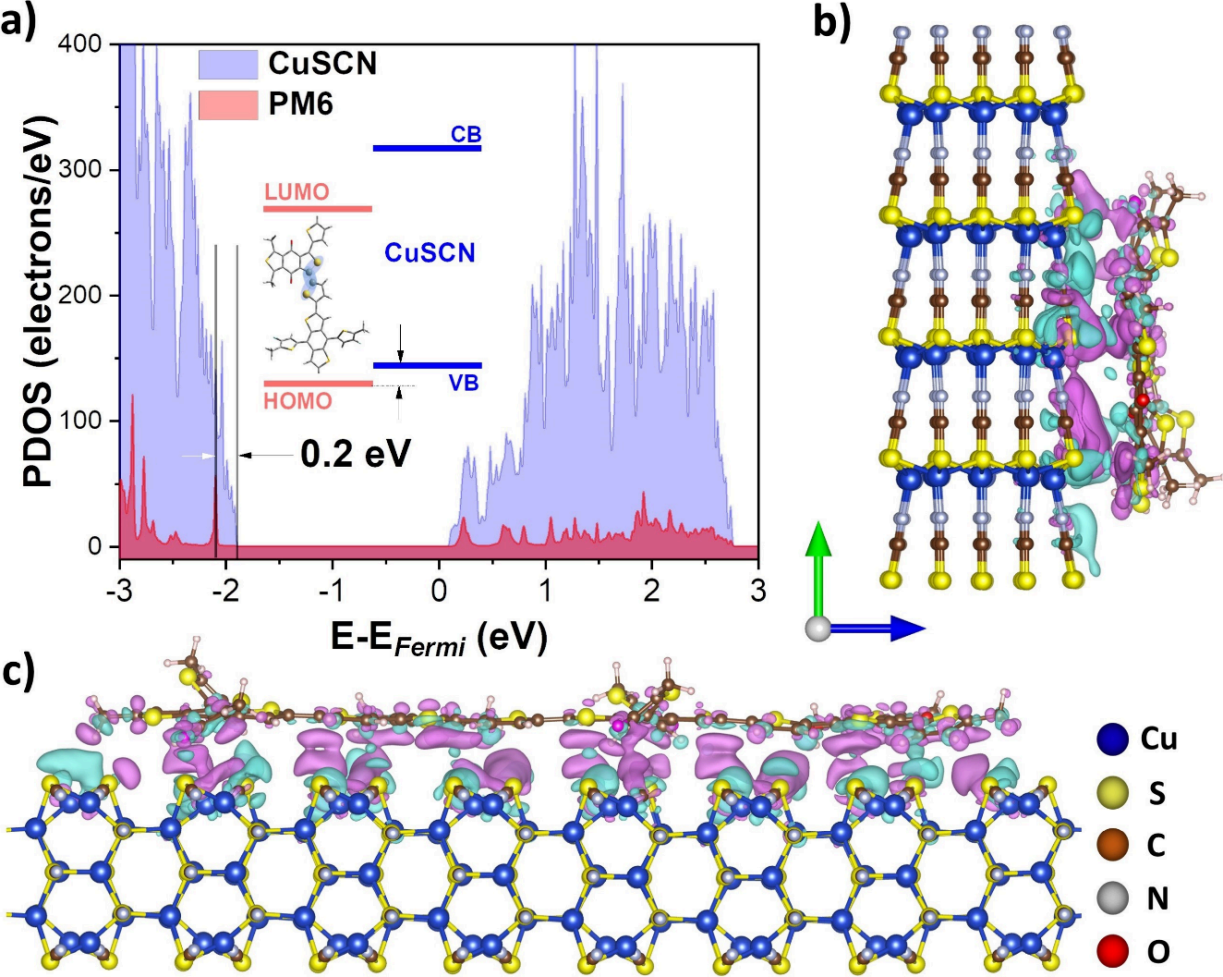
(a) Projected density of states (PDOS) for heterostructure of PM6 adsorbed on the β-CuSCN [112̅0] surface. (b, c) Charge density difference plot when PM6 is adsorbed on the β-CuSCN [112̅0] surface from different perspectives. Turquoise orbitals indicate regions of positive electron density, and purple orbitals indicate regions of negative electron density.

The projected density of states (Figure 5a) reveals that the HOMO level of the organic molecule is slightly below the valence band maximum (VBM) of the CuSCN, and the energy difference between the VBM of the β-CuSCN surface and the HOMO of PM6 is 0.2 eV. This slight energy difference between frontier energy levels can enhance hole transfer at the interface, which is elaborated further in the discussion. Lastly, the calculations reveal that the VBM of the β-CuSCN has a prominent contribution from the Cu d-levels (Figure S12).

The interface between the PM6 and β-CuSCN surface is analyzed as the difference in the charge density between the hybrid and individual components:
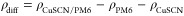
1where ρ_CuSCN/PM6_, ρ_PM6_, and ρ_CuSCN_ denote the charge densities of the total and individual components of PM6 and CuSCN, respectively, and are depicted in Figure 5b,c. The figure reveals a significant charge transfer, quantified by the Bader charge analysis as 0.5e from CuSCN to PM6.^37^ From the optimized structure, the shortest distance between CuSCN and PM6 is 3.2 Å. Because of the considerable charge transfer at the interface and the shorter PM6–CuSCN distance in the optimized geometry, the high electronic coupling between PM6–CuSCN at the interface is highly likely to enhance the charge transfer properties.

## 3 Discussion

The application of femtosecond mid-IR spectroscopy to study and explore the hole injection at the device interface is novel, with access to molecular-level dynamical information. We conclude that the spectral feature at 2180 cm^–1^ in Figure 3d originates from the hole transfer from PM6 and not the direct excitation of CuSCN for the following reasons. First, the experimentally chosen excitation value for PM6 (600 nm) is far from the minimal energy necessary to excite across the bandgap of CuSCN (320 nm). Second, the possibility of two-photon absorption is also ruled out due to the low excitation density and low-absorption cross section of CuSCN. Furthermore, an electron drift is also ruled out, as the energy of the conduction band of CuSCN lies well above the LUMO of PM6. To help rule out a spontaneous hole transfer in the ground state, we obtained the current–voltage characteristic curve in a single-layer solar cell device arrangement of indium tin oxide (ITO)/CuSCN/PM6/perylene diimide (PDINN)/silver (Ag) in dark and illuminated conditions with the PDINN as an electron transport layer and ITO and Ag as the transparent and metal electrodes, respectively (Figure S13). Under dark conditions, zero current was measured, matching the conditions inside the spectrometer setup, where ambient light is kept out during the measurement. It is evident that a small current flow occurs at both positive and negative applied biases, which supports the conclusion that the energy barrier for hole transfer is low. Based on the DFT results in Figure 5a, the energy barrier is estimated to be approximately 0.2 eV. The calculated energy barrier of 0.2 eV between the VBM of the β-CuSCN surface and the HOMO of PM6 precludes the possibility of a thermally assisted hole transfer in the ground state because it lies well above the thermal energy (kT) at room temperature (∼0.025 eV). Lastly, we can also rule out charge transfer to any band-tail states in CuSCN, which have previously been shown to assist with hole injection assuming the correct energy level alignment with an organic photoabsorber.^25^ In the bilayer arrangement, according to our calculations, the HOMO of PM6 aligns within the VB of CuSCN; therefore, we would expect hole transfer into the VB instead of into band-tail states.

According to the time-resolved mid-IR results and associated kinetics (Figure S6), we observed a fast rise within 168 fs for the C=O and CN vibrational marker modes. We assigned the fast relaxation of C=O in early times to the fast vibrational redistribution of carbonyl bonds because they all lie perpendicular to the direction of the main transition dipole vector along the thiophene section of PM6. The difference between the long components is likely due to the difference in dielectric constants between the two materials. Organic polymers in photovoltaics typically have lower dielectric constants; thus, the binding energy between electron–hole pairs (excitons) is higher, leading to faster recombination rates.^38,39^ For the reasons mentioned, the positive signal for CN at 2180 cm^–1^ arises solely due to the hole transfer to CuSCN and represents the change in the CN quantity with a vibrational frequency blue-shifted by the injected positive charges. To the best of our knowledge, the ultrafast nature of the hole injection in the proposed system has never been measured on this time scale, highlighting the effectiveness of the fs mid-IR technique in revealing these dynamics. To aid in the description of the factors necessary for an ultrafast hole injection, we review the literature. Thus far, the literature contains several studies using the fs mid-IR technique to study electron injection dynamics by following the marker modes on both electron donors and acceptors and has successfully elucidated an ultrafast electron transfer on different occasions. In a series of studies by John Asbury et al.,^40^ ultrafast electron injection was observed between several polymer/fullerene acceptor systems. In one such study, after selective excitation of the polymer poly[2-methoxy-5-(2-ethylhexyloxy)-1,4-(1-cyanovinylene-1,4-phenylene)] (CN-MEH-PPV), the kinetics were obtained by following the methyl ester carbonyl bleach signal on the electron acceptor [6,6] phenyl-C_61_-butyric acid methyl ester (PCBM), revealing a fast approximate time component of 90 fs. Other fullerene studies have reported electron transfer times as fast as ∼45 fs.^41^

Although no complete explanation exists for the fast charge transfer times, several principal factors have been proposed. Interfacial distance is crucial, with time scales of up to 1 ps for distances between 4 and 7 Å.^42,43^ From the computational results, the interfacial distance between the two closest points was 3.2 and 3.6 Å between the conjugated thiophene section of the polymer and the protruding sulfur atoms of the CuSCN surface. Thus, the reported time scale for the hole transfer aligns with the expected interfacial distances detailed in the literature. Furthermore, the short interfacial gap between PM6 and CuSCN is likely facilitated by a strong electrostatic attraction following the ground-state charge transfer and consequential interfacial dipole when the two surfaces are initially brought together.

From the TD-DFT calculations, we obtained the transition dipole moment vector of the principal 600 nm transition of PM6 along the conjugated thiophene bridge section. The vector is parallel to the S–Cu bond along the surface of CuSCN, corresponding to the VBM of CuSCN, which primarily contributes to the Cu d-levels. The difference in charge density in Figure 4b,c also indicates the dominant orbital contributions from the Cu atoms to the π molecular orbitals along the thiophene bridge in PM6. Despite the lack of covalent bonding between the two materials, the negative regions transverse the relatively narrow interfacial gap into the CuSCN lattice, indicating delocalization of the PM6 frontier orbitals in the ground state. Therefore, the parallel alignment between the transition dipole moment of PM6 with CuSCN Cu d-orbitals and the wave function delocalization of the surface polarizable π-conjugated PM6 further supports the case for a coherent charge transfer in the proposed system.

We can qualitatively describe the observed ultrafast hole transfer behavior from the kinetic and computational results. Although the free-energy difference between the two energy levels can affect the rate of free carrier formation, it can also influence the initial interfacial charge transfer time.^44^ In this case, charge transfer happens so fast that there is no time to build up a constant photovoltage between the interface and bulk of the photoabsorber (necessary, for instance, to enable continuous solar energy conversion under illumination). Therefore, the instantaneous generation of excitons must establish a pseudo-photovoltage at the interface at the moment of excitation, enabling charges on either side of the interface to overcome the relatively small 0.2 eV potential barrier.^27^ The high degree of coupling between the two systems facilitates an initial coherent charge transfer event without the transfer of thermal energy (adiabatic charge transfer). This type of coherent charge transfer has been observed in several studies, including organic heterojunctions where the interface morphology is expected to be more complex.^45,46^ Going forward, any complete theoretical explanations of charge transfer at these interface types necessitate including coherence effects, which are not fully accounted for in Marcus or Onsager–Braun electron transfer models.^47−49^

A dipole at the interface can cause a change in the vibrational frequencies compared to the bulk (the vibrational Stark effect) observed throughout the pump–probe interval.^40,50,51^ If the shift is large enough, it is possible to observe two separate bands for the same vibrational band, corresponding to bulk and interface populations. We observed no significant shift in the relevant bands over the time scale of the fs mid-IR measurements; however, if the shift is slight, it could be occluded beneath the broader/larger vibrational bands. Furthermore, intraband absorption by IR photons (broad offset in the difference spectrum) in addition to broad polaronic absorption by PM6 may also make it challenging to observe the Stark effect.

## 4 Conclusion

When analyzing the interfacial arrangement of PM6/CuSCN, an ultrafast and coherent hole injection process was unveiled within an estimated 168 fs. This outcome was related to the fast rise of the CN band along with that of the C=O band. Further explorations using a slab model arrangement divulged electronic and morphological details at the interface of the two materials. The report indicates an interfacial minimum distance of 3.2 Å and an insignificant energetic barrier between the CuSCN VBM and PM6 HOMO, further supporting the coherent charge transfer as the prime mechanism behind the ultrafast generation of a CN signal in CuSCN settings.

The findings also highlighted the high coupling regime between the two materials without covalent bonding. For the first time, the study demonstrates the application of femtosecond mid-IR spectroscopy specifically for measuring ultrafast coherent hole transfers in the nascent HTL CuSCN and at an organic/semiconductor interface. The results emphasize the significance of choosing suitable absorber materials to facilitate fast hole transfers. These findings could provide critical insight into researchers in photovoltaic and optoelectronic sectors to enhance CuSCN-type semiconductor performance and their potential incorporation into commercial devices.

## 5 Experimental Methods

### 5.1 Synthesis

A 40 mg/mL concentration of copper(I) thiocyanate (CuSCN; 99%, Sigma-Aldrich) was dissolved in diethyl sulfide (Sigma-Aldrich). The solution was stirred overnight at 50 °C under a N_2_-filled glovebox. Then, the solution was filtered using a 32 mm Acrodisc syringe filter with a 5 μm support membrane. In contrast, PM6 was dissolved in chloroform at a 10 mg/mL concentration. The solution of PM6 was stirred for 1 h at 40 °C in a N_2_-filled glovebox. For the film fabrication, CuSCN and PM6 were deposited on a CaF_2_ window in a N_2_-filled glovebox. Specifically, CuSCN was deposited by spin coating and thermal annealing at 100 °C for 10 min. After the films were cooled to room temperature, PM6 was directly deposited by spin coating without additional treatment.

### 5.2 Steady-State Measurements

Steady-state absorption and photoluminescence (PL) measurements were performed with a Cary 5000 UV–vis spectrometer from Varian and a Fluoromax-4 fluorometer (Horiba), respectively. The FTIR measurements were conducted by using a Cary 600 spectrometer (Agilent). The film samples were placed between two calcium fluoride cell windows for all optical measurements. All of the steady-state measurements of CuSCN, PM6, and the CuSCN/PM6 composite were directly performed on each film.

### 5.3 Time-Resolved Mid-Infrared Spectroscopy

Time-resolved IR experiments were conducted using a Helios-IR spectrometer with a broadband capability (Ultrafast Systems). The pump pulses at 600 nm were obtained by the second harmonic generation of a 120 fs Ti:sapphire regenerative amplifier operating at 1 kHz (Spectra-Physics). The tunable mid-IR probe pulses were generated through difference-frequency mixing in a near-IR optical parametric amplifier (Topas prime-Light Conversion/Spectra-Physics). The experimental setup is detailed elsewhere.^52^ In the transient IR measurements, the photoinduced process was recorded on the PM6, CuSCN, and PM6/CuSCN film samples and placed between two calcium fluoride cell windows. The bilayer PM6/CuSCN structure was excited from the PM6 side, and profilometry measurements revealed that the thickness of the PM6 absorber layer was approximately 60 nm in the pure PM6 and PM6/CuSCN samples. The cell was rotated to ensure that a fresh sample was excited at every laser shot. The spectrometer setup was also covered to remove ambient light.

### 5.4 Computational Methods

The DFT calculations were performed to study the geometries of neutral, radical anion, and excited states of PM6 and the corresponding vibrational modes using restricted and unrestricted B3LYP functionals with the 6-31G(d,p) basis set. Finite-length oligomers with two repeated units represented PM6 to mimic the properties of the corresponding polymer. The geometries and vibrational frequencies of the lowest excited state were obtained through TD-DFT calculations (TD-B3LYP/6-31G(d,p)) for the PM6 oligomer without symmetry constraints.

All calculations were performed using the Gaussian09 program (revision D.01).^53^ Geometry optimization of the pristine slab and PM6 on the slab was performed using the Vienna ab initio simulation package (i.e., VASP) at the generalized gradient approximation level using the Perdew–Burke–Ernzerhof functional.^54^ All calculations were performed with a plane-wave cutoff of 400 eV by using a repeated-slab approach, and the slab was separated by a vacuum space of over 15 Å. In Figure 4, we computed the electronic structure of the supercell cell of the β-CuSCN in the [112̅0] directions with dimensions of 49.79 × 21.91 Å^2^ and an oligomer of PM6 adsorbed on the top surface. The van der Waals dispersion effects were considered at the DFT-D3 level. Monkhorst–Pack *k*-point meshes of 2 × 5 × 1 were considered for the supercell and PM6 oligomer adsorbed on a supercell. During geometry optimizations, all of the atoms of PM6 and atoms of the top two layers of the β-CuSCN were allowed to relax until the atomic forces were smaller than 0.02 eV/Å.

The peaks and intensities for the IR vibrational modes were calculated on the β-CuSCN using the phonon code as implemented in the Quantum Espresso package.^53^ The local density approximation exchange-correlation functional with norm-conserving pseudopotentials was applied based on the optimized natural and charged structures of the CuSCN. The plane-wave expansion cutoff for the wave functions was set at 80 Ry. A uniform grid of 6 × 6 × 2 for the Monkhorst–Pack scheme was used for the *k*-point sampling with a self-consistency threshold of 10^–14^ Ry.

### 5.5 *J*–*V* Curve and Cell Arrangement

Before use, the glass/ITO substrates were cleaned with detergent, acetone, and isopropanol for 10 min each. Then, the substrates were dried and subjected to UV-ozone treatment for 20 min. The deposition of CuSCN and PM6 is the same as mentioned. The PDINN (2 mg/mL in methanol) was spin-coated onto the substrates. Finally, 100 nm of Ag was deposited by thermal evaporation under reduced pressure (<4 × 10^–6^ Pa). The current density–voltage curves were obtained under N_2_ conditions using a Keithley 2400 source meter and an Oriel Class 3A solar simulator calibrated to 1 sun using a silicon reference with a KG5 filter from Newport.

## Supporting Information Available

Tauc plot analysis, PM6/CuSCN *J*–*V* curve, photoelectron spectra, CuSCN XRD pattern, fs mid-IR kinetic and contour plots, GIWAXS figures, and optimized PM6 structures (PDF)
