# Supplementary material for: Ultrafast Coherent Hole Injection at the Interface between CuSCN and Polymer PM6 Using Femtosecond Mid-Infrared Spectroscopy
Source: ACS Appl Mater Interfaces. 2024 Apr 4;17(12):17757–66. doi: 10.1021/acsami.4c01156 (PMC11956001; doi:10.1021/acsami.4c01156)
Supplement: Supplementary file 1 — am4c01156_si_001.pdf [file am4c01156_si_001.pdf]

Supporting Information

Ultrafast Coherent Hole Injection at the Interface between CuSCN and polymer PM6 Using Femtosecond Mid-Infrared Spectroscopy

George Healing[† 1,2], Issatay Nadinov[† 1], Wisnu Tantyo Hadmojo[3], Jun Yin[4], Simil Thomas[1], Osman M. Bakr[2], Husam N. Alshareef[5], Thomas D. Anthopoulos[3] and Omar F. Mohammed[* 1,2]

[1]Advanced Membranes and Porous Materials Center, Division of Physical Science and Engineering, King Abdullah University of Science and Technology, Thuwal 23955-6900, Kingdom of Saudi Arabia

[2]KAUST Catalysis Center, Division of Physical Sciences and Engineering, King Abdullah University of Science and Technology, Thuwal 23955-6900, Kingdom of Saudi Arabia

[3]KAUST Solar Center, Physical Science and Engineering Division, King Abdullah University of Science and Technology (KAUST), Thuwal 23955-6900, Kingdom of Saudi Arabia

[4]Department of Applied Physics, The Hong Kong Polytechnic University, Hung Hom, Kowloon, Hong Kong

[5]Materials Science and Engineering, Physical Science and Engineering Division, King Abdullah University of Science and Technology (KAUST), Thuwal 23955-6900, Kingdom of Saudi Arabia

Corresponding Author:
Omar F. Mohammed – Advanced Membranes and Porous Materials Center and KAUST Catalysis Center, Division of Physical Sciences and Engineering, King Abdullah University of Science and Technology, Thuwal 23955-6900, Kingdom of Saudi Arabia; Orcid.org/0000-0001-8500-1130; Email: omar.abdelsaboor@kaust.edu.sa

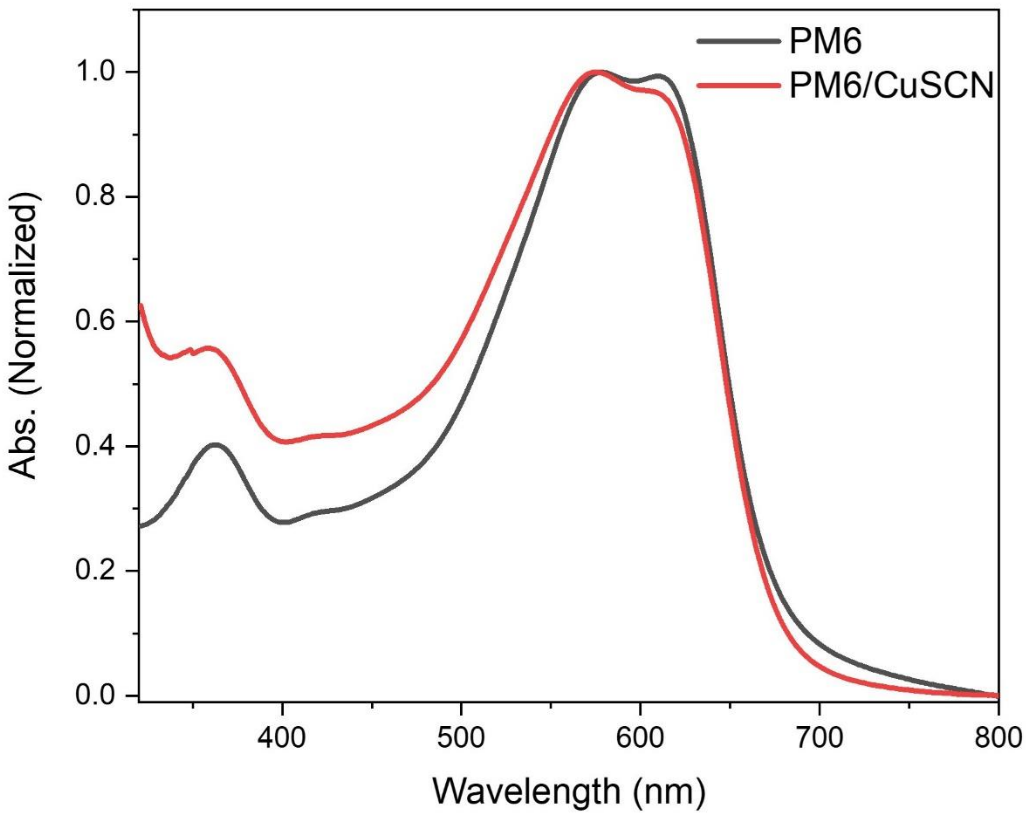

Figure S1. Normalized absorption spectra of PM6 and the PM6/CuSCN bilayer.

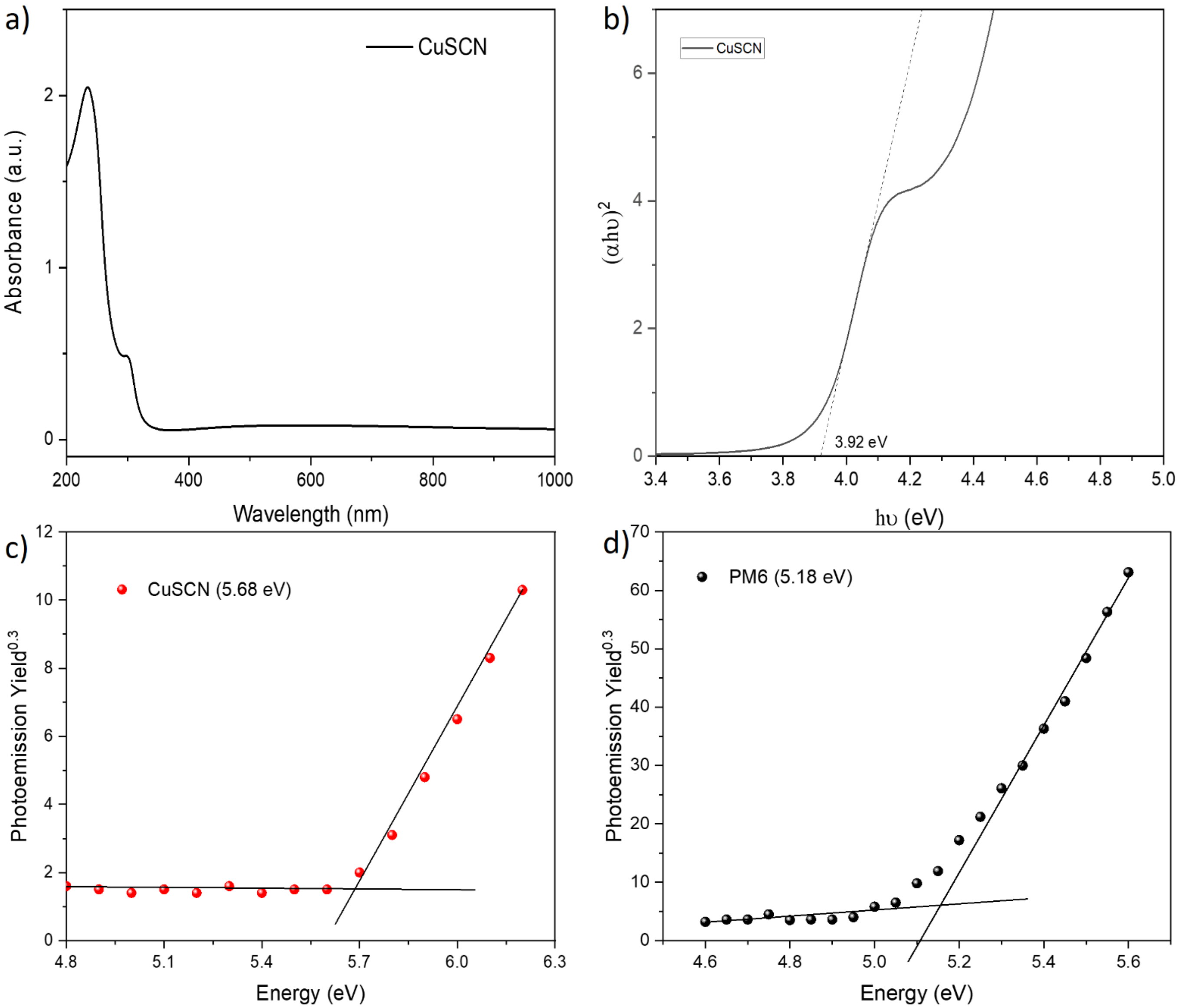

Figure S2. (a) UV-vis absorption spectra of CuSCN on quartz film. (b) Tauc plots calculated from the absorption spectra. (c) and (d) Photoelectron spectroscopy in air (PESA) measurements of CuSCN and PM6 respectively.

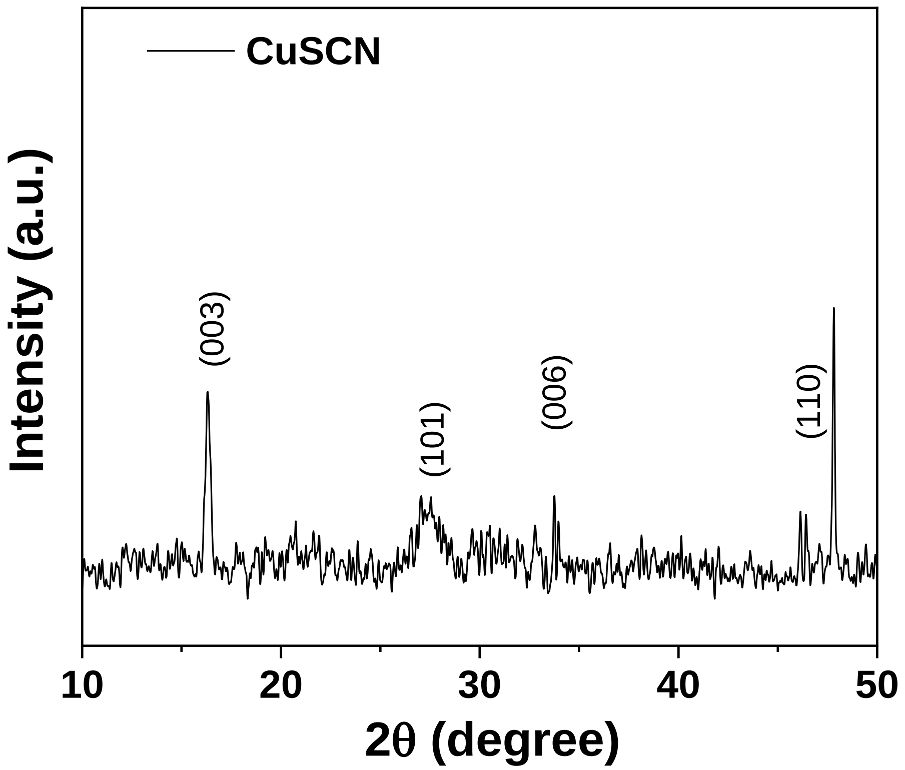

Figure S3. XRD pattern of CuSCN film after annealing at 100 ℃ for 10 min.

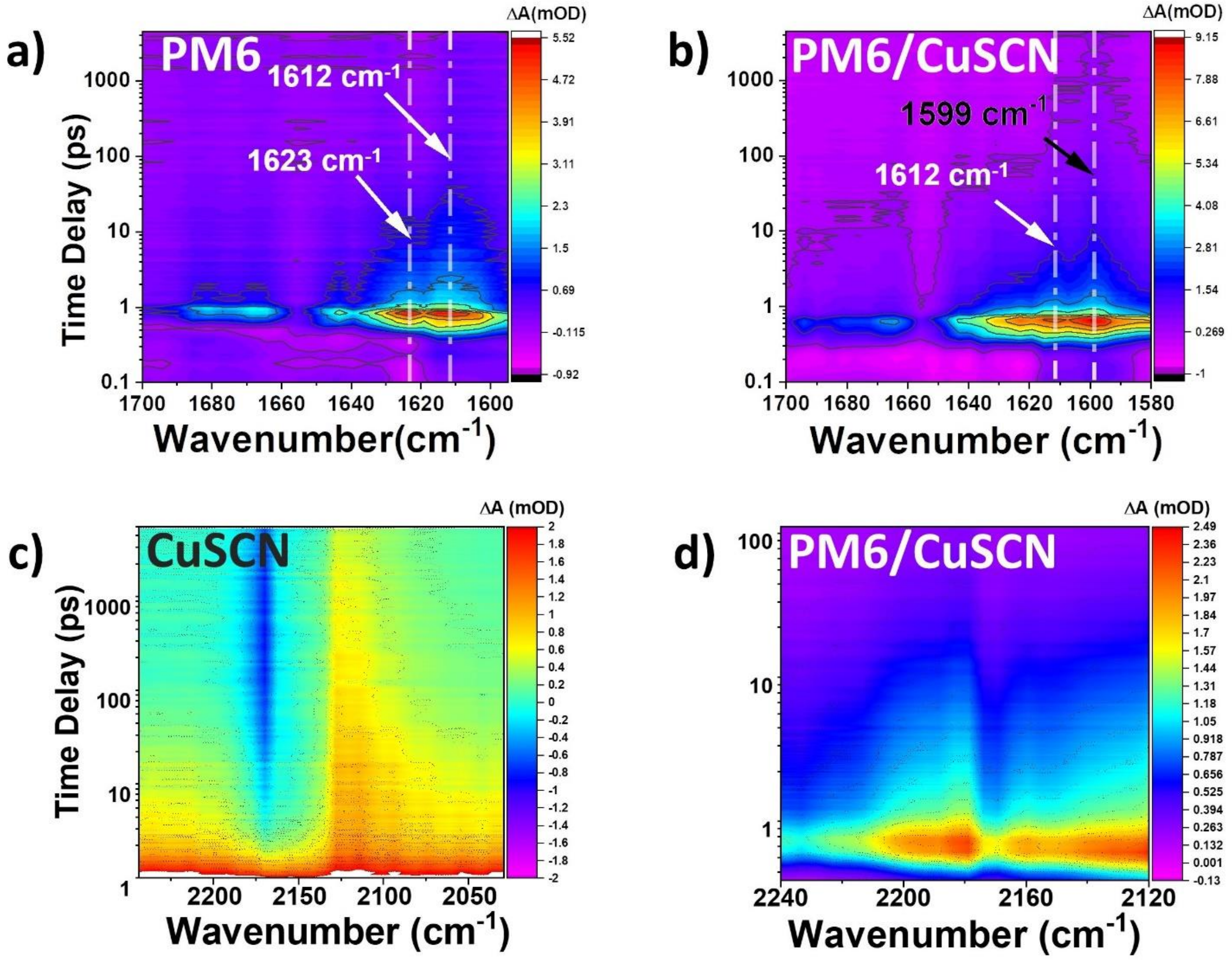

Figure S4. (a,b) Fs mid-infrared (IR) spectroscopy contour plots of PM6 and PM6/CuSCN, probing C=O (λ_{exc}=600 nm). (c,d) fs mid-IR spectra for CuSCN (λ_{exc}=320 nm) and PM6/CuSCN (λ_{exc}=600 nm), respectively, probing the CN stretch region.

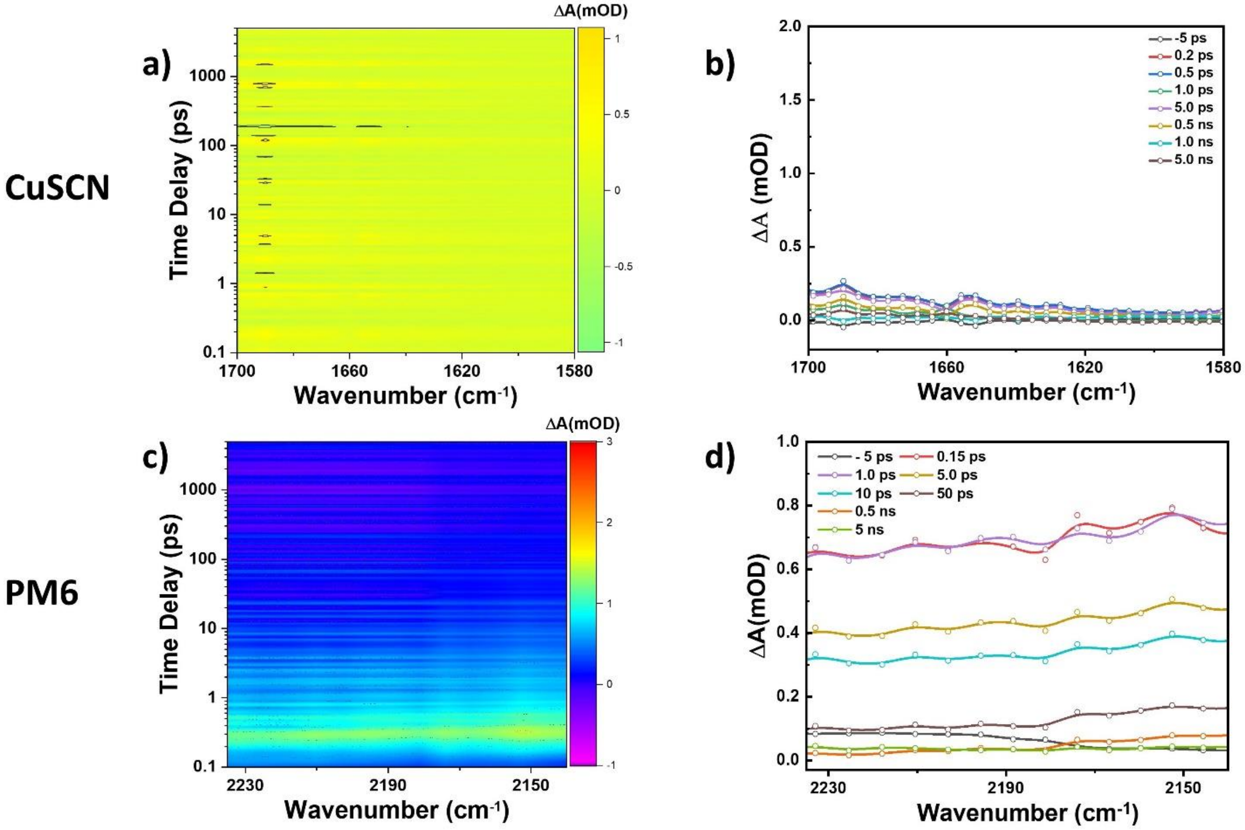

Figure S5. Fs mid-IR spectra and contour plots of the control experiments. (a-b) shows CuSCN probed in the C=O region at ~1640 cm^{-1} (λ_{exc}=320 nm). (c-d) shows PM6 probed in the CN region at ~ 2170 cm^{-1} (λ_{exc}=600 nm).

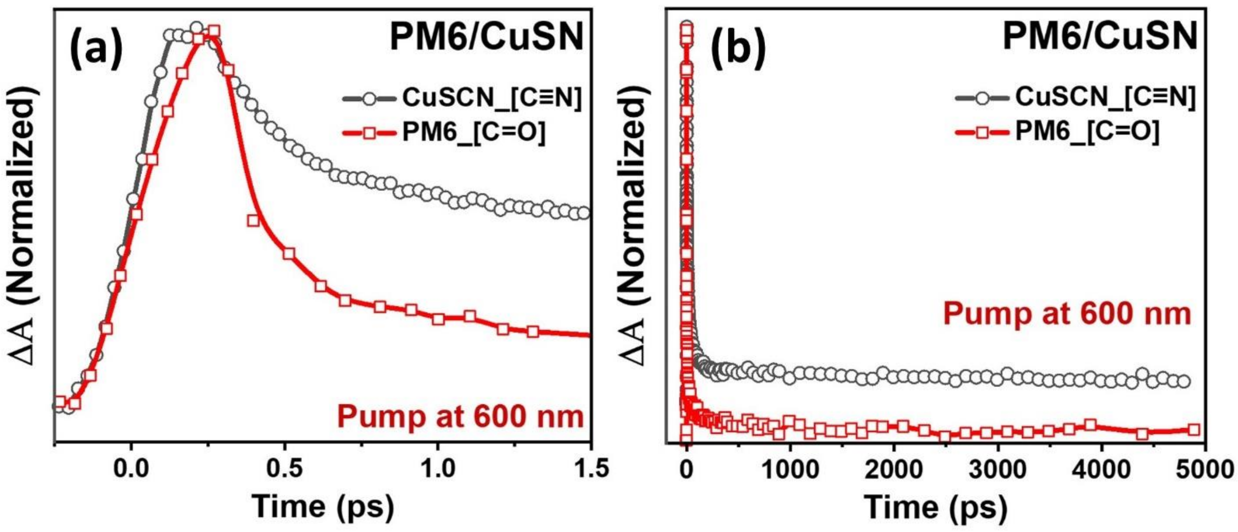

Figure S6. Normalized kinetics plot of CN stretching vibration at 2180 cm^{-1} of CuSCN (black), and C=O carbonyl stretching vibration at 1599 cm^{-1} of PM6 (red) in the PM6/CuSCN system after 600 nm excitation at (a) short and (b) long time ranges.

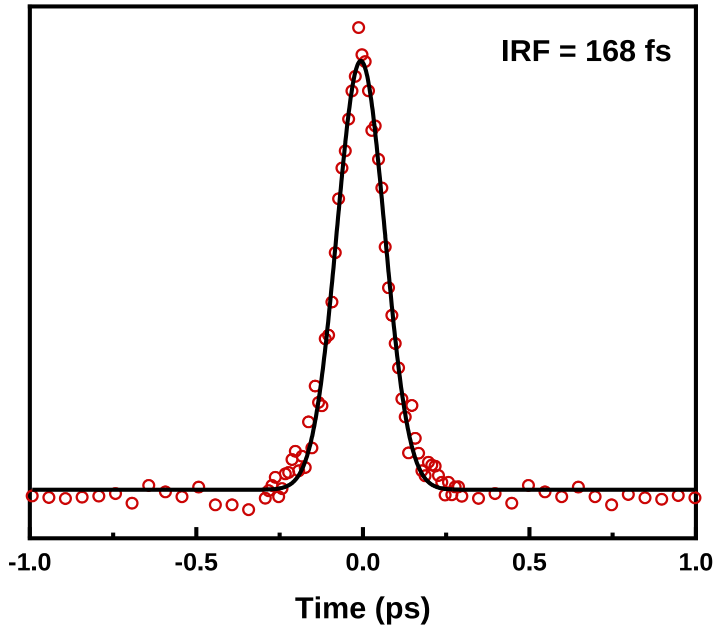

Figure S7. The IRF of the pump pulse in the fs mid-IR set up. A pulse width of 168 fs is obtained by measuring the scattering of methanol.

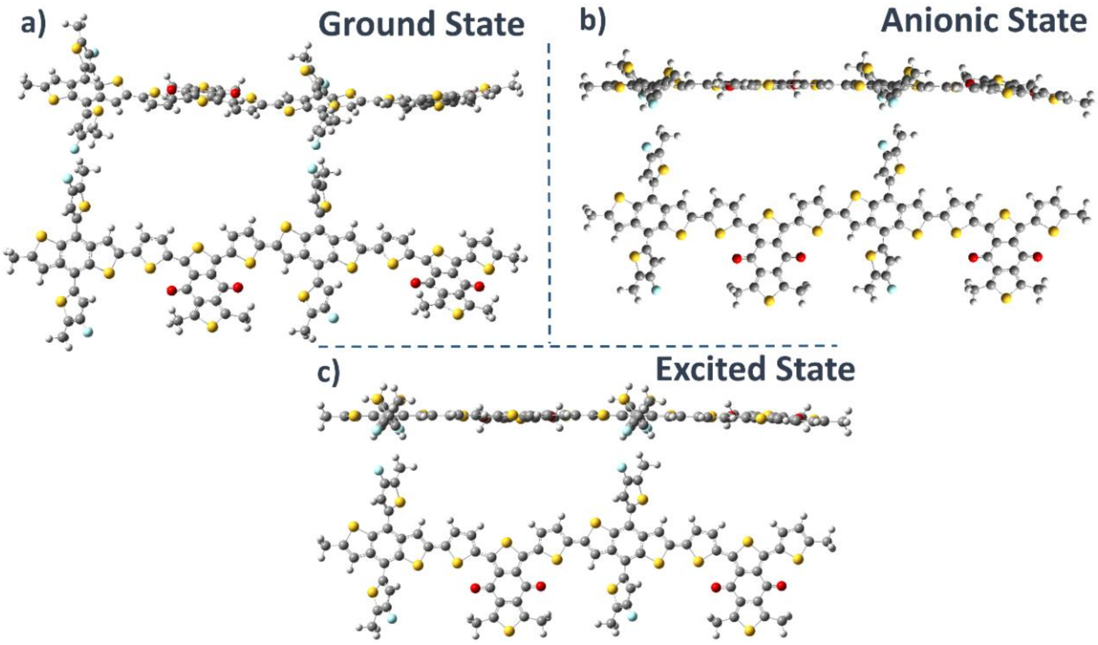

Figure S8. DFT calculated structure for the PM6 polymer chain at (a) ground state, (b) anionic state, and (c) at excited state.

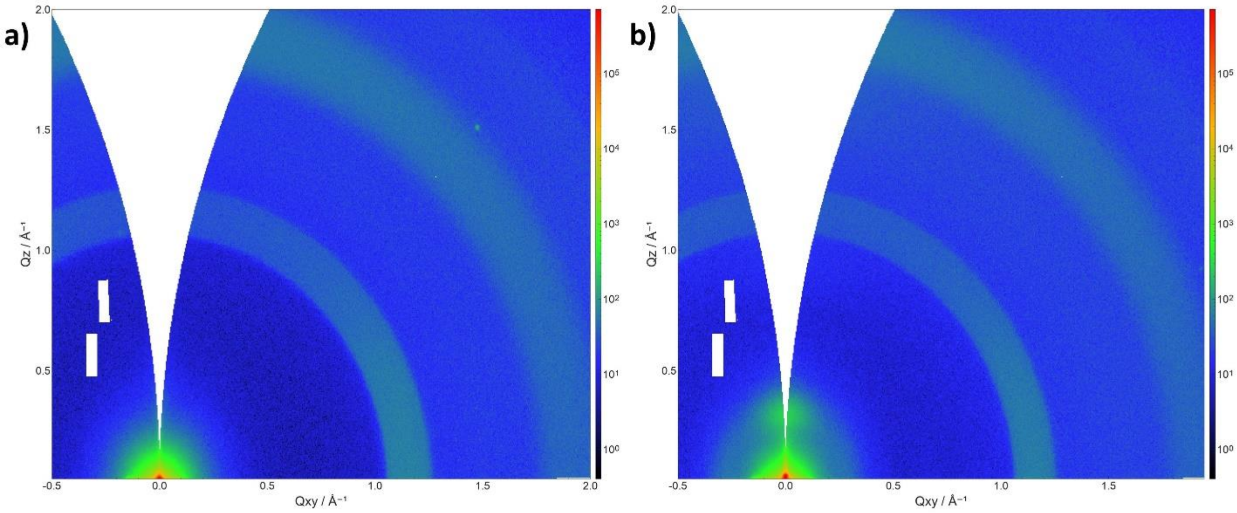

Figure S9. GIWAXS 2d patterns for (a) CuSCN and (b) CuSCN/PM6 films. Instrument: Xenocs Xeuss 3.0 high-resolution (GI)-SAXS/WAXS beamline. Source: Cu 50KeV 0.60mA 1.54189Å. Sample-to-detector-distance: 72mm. Exposure time: 1800s. Incidence angle: 0.3 for CuSCN, 0.4 for CuSCN/PM6.

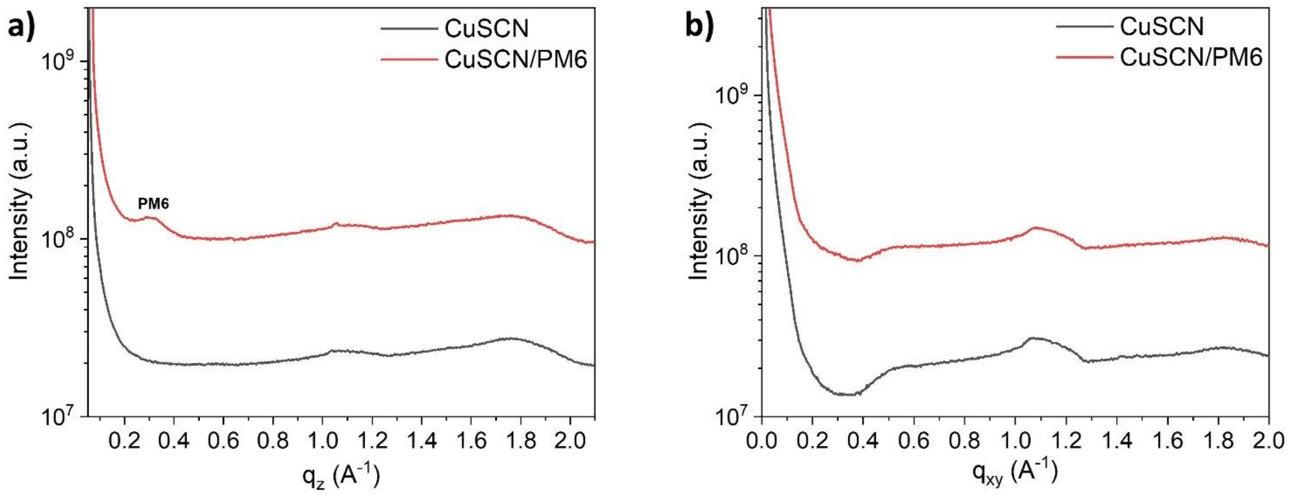

Figure S10. GIWAXS 1D (a) Out-of-plane and (b) In-plane line cut profiles.

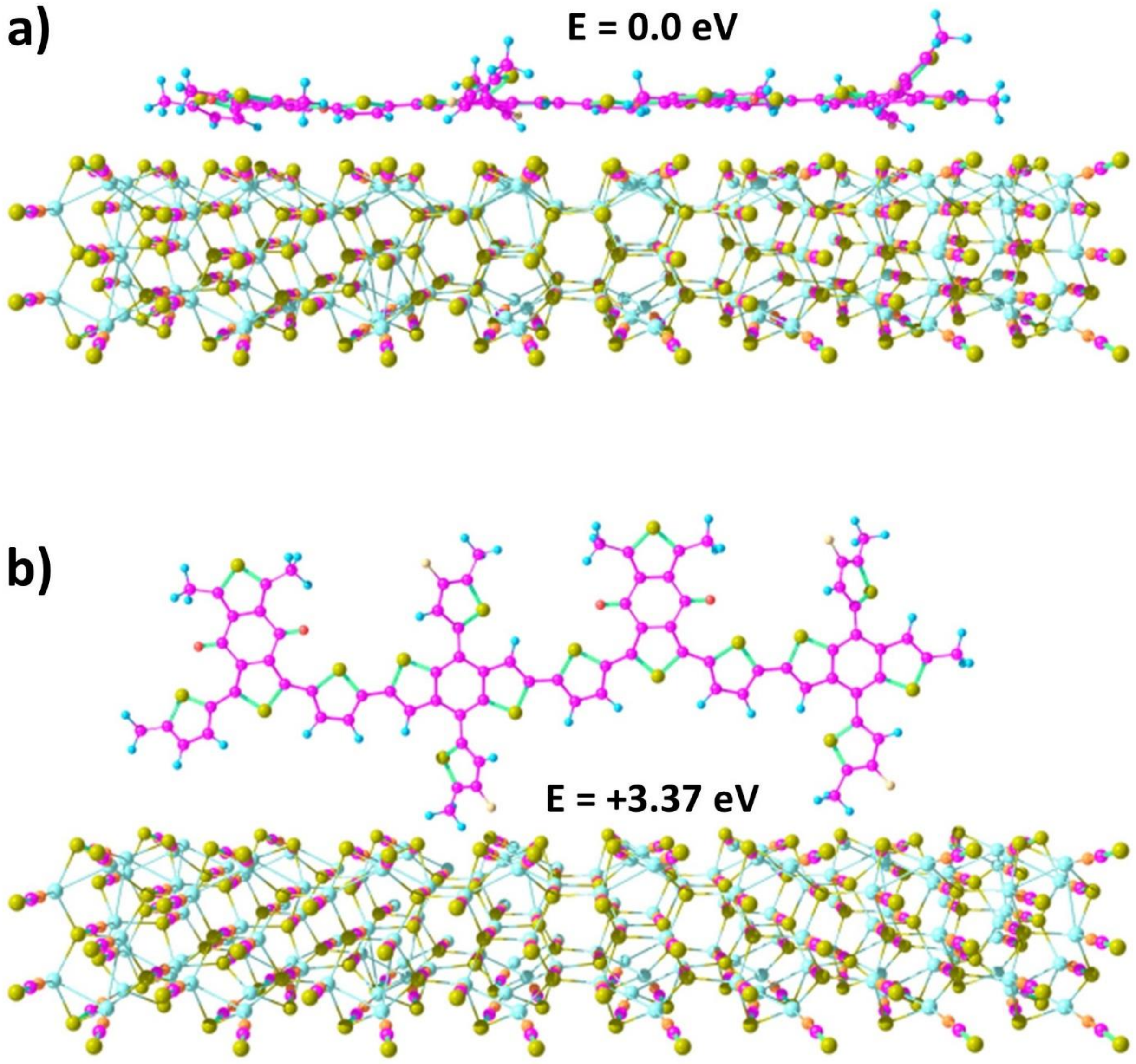

Figure S11. DFT calculated structures for the PM6 polymer on a β-CuSCN using a slab model. (a) PM6 oriented face-on, (b) PM6 oriented side-on.

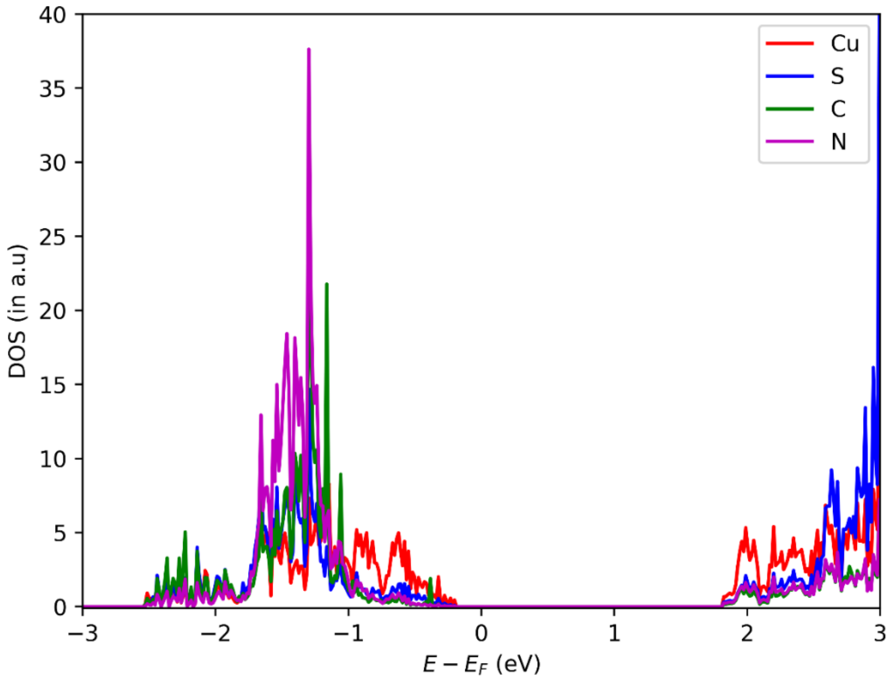

Figure S12. Density of states projected onto Copper (Cu), Sulfur (S), Carbon (C), Nitrogen (N) atoms for heterostructure of PM6 adsorbed on the β-CuSCN [11$\bar{2}$0] surface.

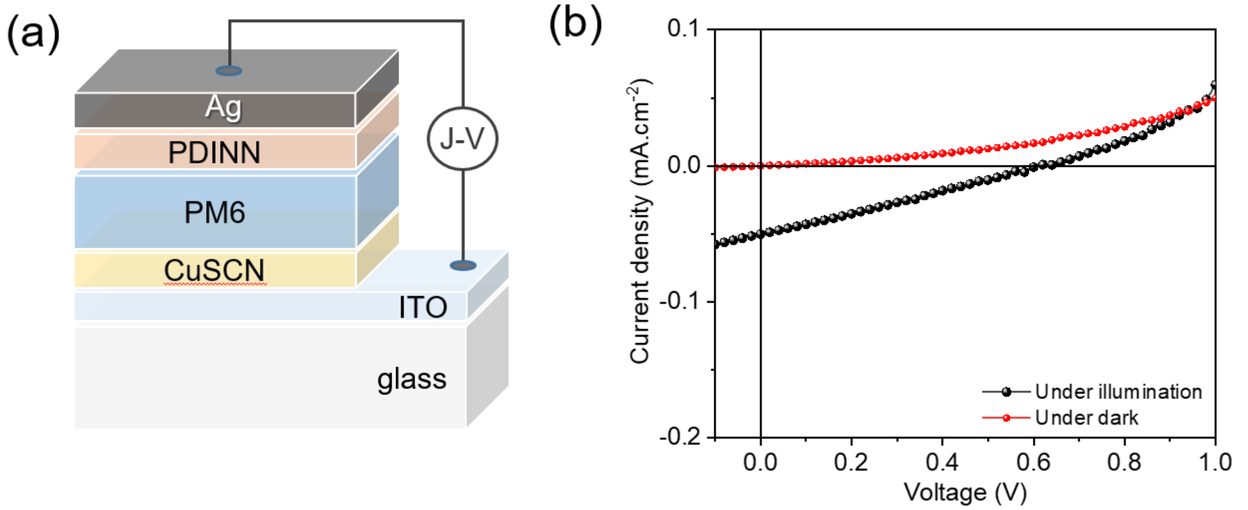

Figure S13. (a) Device structure of single layer organic solar cell. (b) Current density-voltage curves of devices under illumination and dark.
